# *O*,*O*-Dimethyl *O*-(4-sulfamoylphen­yl) phospho­rothio­ate (cythio­ate)

**DOI:** 10.1107/S1600536813014724

**Published:** 2013-06-08

**Authors:** Puhan Zhao, Russell G. Baughman

**Affiliations:** a Department of Chemistry, Truman State University, Kirksville, MO 63501-4221, USA

## Abstract

The title mol­ecule, C_8_H_12_NO_5_PS_2_, exhibits a crystallographic mirror plane that is perpendicular to the ring and bis­ects the sulfamoyl and thio­phosphate ester groups. In the crystal, mol­ecules are linked by N—H⋯O hydrogen-bonding inter­actions reminiscent of carb­oxy­lic acid hydrogen bonding pairs, forming chains parallel to the *b-*axis direction.

## Related literature

The structure of a very similar compound {Famphur; systematic name *O*,*O*-dimethyl *O*-[*p*-[(*N*,*N*-dimethylsulfamoyl)phenyl] phosphorothioate} was published by Baughman (1997 ▶).
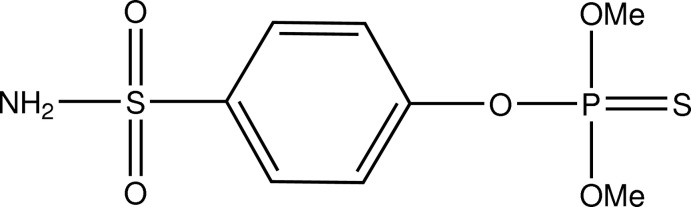


## Experimental

### 

#### Crystal data

C_8_H_12_NO_5_PS_2_
*M* *_r_* = 297.28
Monoclinic, 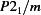

*a* = 6.6634 (2) Å
*b* = 8.5514 (3) Å
*c* = 11.8716 (5) Å
β = 105.067 (3)°
*V* = 653.21 (4) Å^3^
*Z* = 2
Mo *K*α radiation
μ = 0.54 mm^−1^
*T* = 295 K
0.50 × 0.38 × 0.26 mm

#### Data collection

Bruker P4 diffractometer
Absorption correction: integration (*XSHELL*; Bruker, 1999 ▶) *T*_min_ = 0.745, *T*_max_ = 0.890
2229 measured reflections
1591 independent reflections
1421 reflections with *I* > 2σ(*I*)
*R*_int_ = 0.031
3 standard reflections every 100 reflections intensity decay: 1.2%

#### Refinement

*R*[*F* ^2^ > 2σ(*F* ^2^)] = 0.041
*wR*(*F* ^2^) = 0.112
*S* = 1.21
1591 reflections
89 parameters
5 restraints
H-atom parameters constrained
Δρ_max_ = 0.38 e Å^−3^
Δρ_min_ = −0.30 e Å^−3^

### 

Data collection: *XSCANS* (Bruker, 1996 ▶); cell refinement: *XSCANS*; data reduction: *XSCANS*; program(s) used to solve structure: *SHELXS86* (Sheldrick, 2008 ▶); program(s) used to refine structure: *SHELXL97* (Sheldrick, 2008 ▶); molecular graphics: *SHELXTL/PC* (Sheldrick, 2008 ▶); software used to prepare material for publication: *SHELXTL/PC* and *SHELXL97*.

## Supplementary Material

Crystal structure: contains datablock(s) I, global. DOI: 10.1107/S1600536813014724/rz5064sup1.cif

Structure factors: contains datablock(s) I. DOI: 10.1107/S1600536813014724/rz5064Isup2.hkl

Supplementary material file. DOI: 10.1107/S1600536813014724/rz5064Isup3.cml

Additional supplementary materials:  crystallographic information; 3D view; checkCIF report

## Figures and Tables

**Table 1:** Hydrogen-bond geometry (Å, °)

*D*—H⋯*A*	*D*—H	H⋯*A*	*D*⋯*A*	*D*—H⋯*A*
N1—H1⋯O3^i^	0.86	2.36	3.134 (3)	150

Symmetry code: (i) 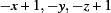.

**Fig. 1.**
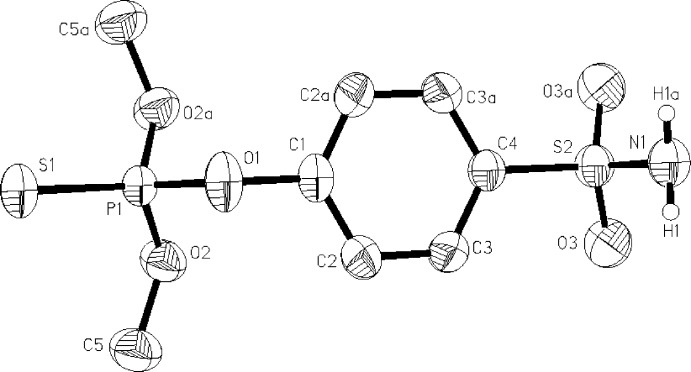
One molecule (2 asymmetric units) of the title compound showing the labeling of all atoms. For clarity methyl and aryl H's have been omitted. Displacement ellipsoids are drawn at the 50% probability level; H's are drawn as small spheres of arbitrary radius. Atoms labeled with the suffix a are generated by the symmetry operation (x, 1/2-y, z).

**Fig. 2.**
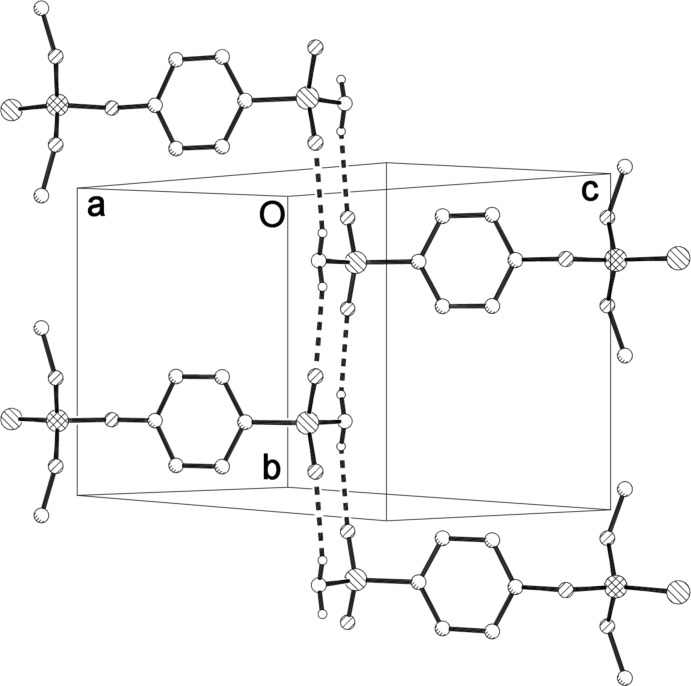
Four molecules (8 asymmetric units) illustrating packing in the b direction. Spheres of arbitrary size are shown. The upper right molecule contains the original asymmetric unit (x,y,z); lower right = (1+x, y, z); upper left = (1-x, -y, 1-z); lower left = (1-x, + y, 1-z). Hydrogen atoms not involved in hydrogen bonding are omitted.

## supplementary crystallographic information

### Comment

Cythioate (Fig. 1) is an organophosphorous insecticide and anthelmintic that has also been sold as Cyflea or Proban. It can be taken orally by pets against fleas, and functions as an acetylcholinesterase inhibitor which interferes with neuromuscular transmission in ectoparastites.

Existence of intermolecular carboxylic acid-like hydrogen bonding is observed between inversion-related H1 and O3 pairs (Fig. 2, Table 1) resulting in the formation of chains parallel to the *b* axis. This hydrogen bonding may also contribute to the observed pyramidal shape on the nitrogen.

Though Cythioate has a structure similar to that of Famphur (*O,O*-dimethyl *O*-[*p*-[(*N,N*-dimethylsulfamoyl)phenyl] phosphorothioate; Baughman, 1997), the differences cannot be ignored as they may be the cause of differences in toxicity/activity. The Cythioate molecule exhibits a mirror plane that is perpendicular to the ring and bisects the sulfamoyl and thiophosphate groups. The presence of this mirror and the absence of one in Famphur result from most of the differences in overall molecular shape as well as specific differences.

Between Cythioate and Famphur a 70 σ difference in the O2—P—S1 bond angles, a 60 σ difference in the O2—P—O2a angles, and a 65 σ difference in O1—P—S1 bond angles are observed. This may be due to the distortion of the phosphorothioate group caused by the hydrogen bonding in Famphur. Interestingly, the P1—O1, P1—O2, and P1—S1 bond lengths are within 3 σ of each other.

### Experimental

Crystals were grown by slow evaporation of a solution in EtOH.

### Refinement

Approximate positions of the six symmetry-independent H's were first obtained from a difference map, then placed into "ideal" positions and refined as a rotational group. Bond lengths were constrained at 0.93 Å (AFIX 43) for aryl C—H's at 0.96 Å (AFIX 137) for methyl C—H's; and at 0.86 Å (DFIX s: N1—H1 = 0.86 Å; H1···H1A = 1.404 Å ; S2···H1 = 2.092 Å to insure that H1–N1–H1A = 109.5°) for N—H's. *U*_iso_(H) were fixed at 1.5*U*_eq_(parent) for NH and methyl H's, and 1.2 *U*_eq_(parent) for all other H's.

In the final stages of refinement, two reflections with very small or negative *F_o_*'s were deemed to be in high disagreement with their *F_c_*'s and were eliminated from final refinement.

### Crystal data

**Table:**

C_8_H_12_NO_5_PS_2_	*F*(000) = 308
*M**_r_* = 297.28	*D*_x_ = 1.511 Mg m^−^^3^
Monoclinic, *P*2_1_/*m*	Mo *K*α radiation, λ = 0.71073 Å
Hall symbol: -P 2yb	Cell parameters from 100 reflections
*a* = 6.6634 (2) Å	θ = 11.3–20.7°
*b* = 8.5514 (3) Å	µ = 0.54 mm^−^^1^
*c* = 11.8716 (5) Å	*T* = 295 K
β = 105.067 (3)°	Parallelepiped, colorless
*V* = 653.21 (4) Å^3^	0.50 × 0.38 × 0.26 mm
*Z* = 2	

### Data collection

**Table:**

Bruker P4 diffractometer	1421 reflections with *I* > 2σ(*I*)
Radiation source: normal-focus sealed tube	*R*_int_ = 0.031
Graphite monochromator	θ_max_ = 27.5°, θ_min_ = 1.8°
θ/2θ scans	*h* = −1→8
Absorption correction: integration (*XSHELL*; Bruker, 1999)	*k* = −1→11
*T*_min_ = 0.745, *T*_max_ = 0.890	*l* = −15→15
2229 measured reflections	3 standard reflections every 100 reflections
1591 independent reflections	intensity decay: 1.2%

### Refinement

**Table:**

Refinement on *F*^2^	Secondary atom site location: difference Fourier map
Least-squares matrix: full	Hydrogen site location: inferred from neighbouring sites
*R*[*F*^2^ > 2σ(*F*^2^)] = 0.041	H-atom parameters constrained
*wR*(*F*^2^) = 0.112	*w* = 1/[σ^2^(*F*_o_^2^) + (0.0241*P*)^2^ + 0.6341*P*] where *P* = (*F*_o_^2^ + 2*F*_c_^2^)/3
*S* = 1.21	(Δ/σ)_max_ < 0.001
1591 reflections	Δρ_max_ = 0.38 e Å^−^^3^
89 parameters	Δρ_min_ = −0.30 e Å^−^^3^
5 restraints	Extinction correction: *SHELXL97* (Sheldrick, 2008), Fc^*^=kFc[1+0.001xFc^2^λ^3^/sin(2θ)]^-1/4^
Primary atom site location: structure-invariant direct methods	Extinction coefficient: 0.024 (3)

### Special details

**Table:**

Geometry. All s.u.'s (except the s.u. in the dihedral angle between two l.s. planes) are estimated using the full covariance matrix. The cell s.u.'s are taken into account individually in the estimation of s.u.'s in distances, angles and torsion angles; correlations between s.u.'s in cell parameters are only used when they are defined by crystal symmetry. An approximate (isotropic) treatment of cell s.u.'s is used for estimating s.u.'s involving l.s. planes.
Refinement. Refinement of *F*^2^ against ALL reflections. The weighted *R*-factor, *wR*, and goodness of fit, *S*, are based on *F*^2^, conventional *R*-factors, *R*, are based on *F*, with *F* set to zero for negative *F*^2^. The threshold expression of *F*^2^ > 2σ(*F*^2^) is used only for calculating *R*-factors(gt) *etc*. and is not relevant to the choice of reflections for refinement. *R*-factors based on *F*^2^ are statistically about twice as large as those based on *F*, and *R*-factors based on ALL data will be even larger.

### Fractional atomic coordinates and isotropic or equivalent isotropic displacement parameters (Å^2^)

**Table:**

	*x*	*y*	*z*	*U*_iso_*/*U*_eq_	
S1	−0.23323 (18)	0.2500	1.05126 (9)	0.0538 (3)	
S2	0.35491 (5)	0.2500	0.47670 (7)	0.0436 (3)	
P1	−0.21467 (14)	0.2500	0.89334 (8)	0.0369 (3)	
O1	0.0232 (4)	0.2500	0.8914 (2)	0.0492 (7)	
O2	−0.3172 (3)	0.1128 (2)	0.81215 (17)	0.0516 (5)	
O3	0.3035 (3)	0.1053 (3)	0.41556 (17)	0.0604 (6)	
N1	0.60337 (16)	0.2500	0.5300 (2)	0.0517 (8)	
H1	0.6458	0.1679	0.5711	0.078*	
C1	0.0859 (5)	0.2500	0.7865 (3)	0.0394 (8)	
C2	0.1203 (5)	0.1098 (4)	0.7397 (3)	0.0521 (7)	
H2	0.0929	0.0163	0.7727	0.063*	
C3	0.1974 (4)	0.1100 (4)	0.6413 (3)	0.0498 (7)	
H3	0.2222	0.0160	0.6078	0.060*	
C4	0.2367 (5)	0.2500	0.5937 (3)	0.0376 (7)	
C5	−0.3293 (6)	−0.0430 (4)	0.8543 (3)	0.0646 (9)	
H5A	−0.3981	−0.1095	0.7908	0.097*	
H5B	−0.1916	−0.0817	0.8881	0.097*	
H5C	−0.4061	−0.0423	0.9125	0.097*	

### Atomic displacement parameters (Å^2^)

**Table:**

	*U*^11^	*U*^22^	*U*^33^	*U*^12^	*U*^13^	*U*^23^
S1	0.0682 (7)	0.0611 (7)	0.0411 (5)	0.000	0.0304 (5)	0.000
S2	0.0563 (6)	0.0460 (5)	0.0327 (4)	0.000	0.0191 (4)	0.000
P1	0.0415 (5)	0.0381 (5)	0.0349 (4)	0.000	0.0167 (4)	0.000
O1	0.0391 (13)	0.077 (2)	0.0339 (13)	0.000	0.0138 (10)	0.000
O2	0.0653 (12)	0.0450 (11)	0.0449 (10)	−0.0096 (9)	0.0150 (9)	−0.0015 (9)
O3	0.0803 (14)	0.0615 (14)	0.0433 (10)	−0.0078 (12)	0.0233 (10)	−0.0151 (10)
N1	0.056 (2)	0.055 (2)	0.0513 (19)	0.000	0.0257 (16)	0.000
C1	0.0340 (17)	0.053 (2)	0.0332 (16)	0.000	0.0125 (13)	0.000
C2	0.0647 (17)	0.0463 (16)	0.0550 (16)	0.0077 (14)	0.0330 (14)	0.0131 (13)
C3	0.0656 (17)	0.0399 (15)	0.0527 (15)	0.0062 (13)	0.0313 (13)	0.0026 (12)
C4	0.0394 (17)	0.0416 (19)	0.0329 (16)	0.000	0.0118 (13)	0.000
C5	0.091 (2)	0.0439 (17)	0.0617 (19)	−0.0109 (17)	0.0243 (17)	−0.0017 (15)

### Geometric parameters (Å, º)

**Table:**

S1—P1	1.9107 (13)	C1—C2	1.366 (3)
S2—O3^i^	1.431 (2)	C1—C2^i^	1.366 (3)
S2—O3	1.431 (2)	C2—C3	1.393 (4)
S2—N1	1.6114 (6)	C2—H2	0.9300
S2—C4	1.766 (3)	C3—C4	1.377 (3)
P1—O2^i^	1.559 (2)	C3—H3	0.9300
P1—O2	1.559 (2)	C4—C3^i^	1.377 (3)
P1—O1	1.591 (3)	C5—H5A	0.9604
O1—C1	1.413 (4)	C5—H5B	0.9599
O2—C5	1.433 (4)	C5—H5C	0.9606
N1—H1	0.8600		
			
O3^i^—S2—O3	119.80 (19)	C2^i^—C1—O1	118.57 (16)
O3^i^—S2—N1	106.56 (11)	C1—C2—C3	118.5 (3)
O3—S2—N1	106.56 (11)	C1—C2—H2	120.7
O3^i^—S2—C4	107.59 (10)	C3—C2—H2	120.7
O3—S2—C4	107.59 (10)	C4—C3—C2	119.7 (3)
N1—S2—C4	108.30 (16)	C4—C3—H3	120.1
O2^i^—P1—O2	97.65 (16)	C2—C3—H3	120.1
O2^i^—P1—O1	105.61 (10)	C3—C4—C3^i^	120.7 (3)
O2—P1—O1	105.61 (10)	C3—C4—S2	119.57 (16)
O2^i^—P1—S1	118.48 (8)	C3^i^—C4—S2	119.57 (16)
O2—P1—S1	118.48 (8)	O2—C5—H5A	109.5
O1—P1—S1	109.46 (11)	O2—C5—H5B	109.4
C1—O1—P1	122.5 (2)	H5A—C5—H5B	109.5
C5—O2—P1	122.63 (19)	O2—C5—H5C	109.5
S2—N1—H1	112.0	H5A—C5—H5C	109.5
C2—C1—C2^i^	122.8 (3)	H5B—C5—H5C	109.4
C2—C1—O1	118.57 (16)		
			
O2^i^—P1—O1—C1	−51.40 (8)	C1—C2—C3—C4	−0.1 (5)
O2—P1—O1—C1	51.40 (8)	C2—C3—C4—C3^i^	−1.1 (6)
S1—P1—O1—C1	180.000 (1)	C2—C3—C4—S2	174.7 (2)
O2^i^—P1—O2—C5	−160.9 (2)	O3^i^—S2—C4—C3	157.3 (3)
O1—P1—O2—C5	90.5 (3)	O3—S2—C4—C3	26.9 (3)
S1—P1—O2—C5	−32.6 (3)	N1—S2—C4—C3	−87.9 (3)
P1—O1—C1—C2	−91.7 (3)	O3^i^—S2—C4—C3^i^	−26.9 (3)
P1—O1—C1—C2^i^	91.7 (3)	O3—S2—C4—C3^i^	−157.3 (3)
C2^i^—C1—C2—C3	1.2 (6)	N1—S2—C4—C3^i^	87.9 (3)
O1—C1—C2—C3	−175.2 (3)		

Symmetry code: (i) *x*, −*y*+1/2, *z*.

### Hydrogen-bond geometry (Å, º)

**Table:**

*D*—H···*A*	*D*—H	H···*A*	*D*···*A*	*D*—H···*A*
N1—H1···O3^ii^	0.86	2.36	3.134 (3)	150

Symmetry code: (ii) −*x*+1, −*y*, −*z*+1.
